# PAReTT: A Python Package for the Automated Retrieval and Management of Divergence Time Data from the TimeTree Resource for Downstream Analyses

**DOI:** 10.1007/s00239-023-10106-3

**Published:** 2023-04-20

**Authors:** Louis-Stéphane Le Clercq, Antoinette Kotzé, J. Paul Grobler, Desiré Lee Dalton

**Affiliations:** 1 South African National Biodiversity Institute, Pretoria, 0001 South Africa; 2 Department of Genetics, University of the Free State, Bloemfontein, 9300 South Africa; 3 School of Health and Life Sciences, Teesside University, Middlesbrough, TS1 3BA UK

**Keywords:** PAReTT, PYTHON, Time trees, Divergence time, Timelines, Diversification rate

## Abstract

Evolutionary processes happen gradually over time and are, thus, considered time dependent. In addition, several evolutionary processes are either adaptations to local habitats or changing habitats, otherwise restricted thereby. Since evolutionary processes driving speciation take place within the landscape of environmental and temporal bounds, several published studies have aimed at providing accurate, fossil-calibrated, estimates of the divergence times of both extant and extinct species. Correct calibration is critical towards attributing evolutionary adaptations and speciation both to the time and paleogeography that contributed to it. Data from more than 4000 studies and nearly 1,50,000 species are available from a central TimeTree resource and provide opportunities of retrieving divergence times, evolutionary timelines, and time trees in various formats for most vertebrates. These data greatly enhance the ability of researchers to investigate evolution. However, there is limited functionality when studying lists of species that require batch retrieval. To overcome this, a PYTHON package termed Python-Automated Retrieval of TimeTree data (PAReTT) was created to facilitate a biologist-friendly interaction with the TimeTree resource. Here, we illustrate the use of the package through three examples that includes the use of timeline data, time tree data, and divergence time data. Furthermore, PAReTT was previously used in a meta-analysis of candidate genes to illustrate the relationship between divergence times and candidate genes of migration. The PAReTT package is available for download from GitHub or as a pre-compiled Windows executable, with extensive documentation on the package available on GitHub wiki pages regarding dependencies, installation, and implementation of the various functions.

**Supplementary Information:**

The online version contains supplementary material available at 10.1007/s00239-023-10106-3.

## Introduction

Evolutionary processes are linked to time, be it diversification within a lineage which may lead to the emergence of a new species, or via subtle molecular changes over several generations steadily driving phenotypic variation (Wagner 2018; Francisco Henao Diaz et al. 2019), leading to speciation at the subspecies or ecotype level. For example, some primary divisions between entire taxonomic orders of birds arose approximately 75 million years ago while more recent divisions between subspecies occurred as recently as 1 million years ago (Prum et al. 2015; Mwale et al. 2022).

**Fig. 1:**
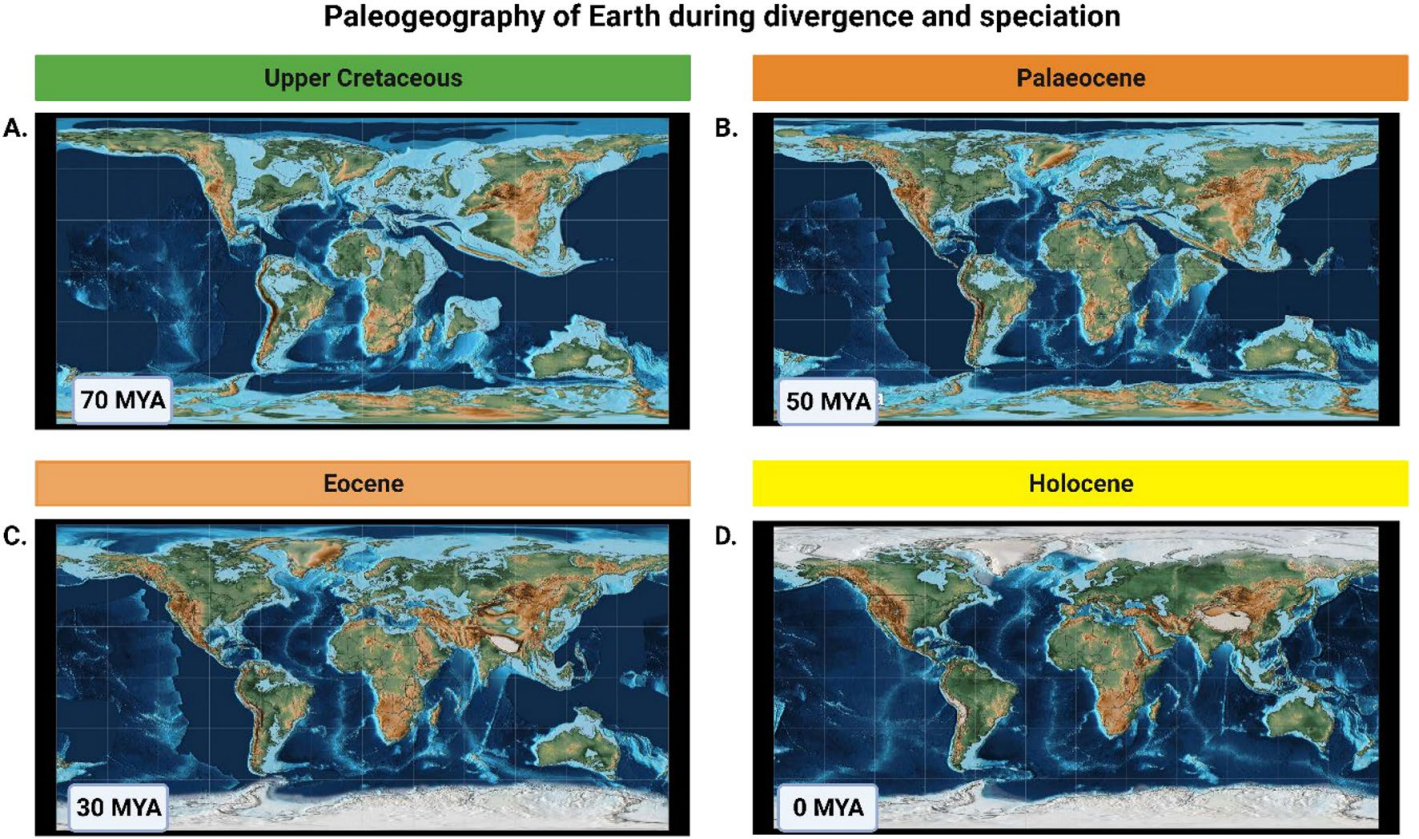
Paleogeographic reconstructions of Earth for the past 70 million years to illustrate environmental differences during the period of divergence using PALEOMAP. **a** Positions of the continents approximately 70 million years ago during the Upper Cretaceous before West Africa had merged with the main continent and when India was still an island. **b** Continents approximately 50 million years ago during the Palaeocene after the African continent formed but before the Americas were connected and shortly before India merged with Asia or the polar caps formed. **c** Geography of Earth during the Eocene, 30 million years ago, by which time most continents had formed but central America did not connect the North and South yet and much of Europe was still under water. **d** Modern day geography of Earth in the current Holocene. (Image created in BioRender.com)

Most evolutionary processes are context dependent and are, thus, studied as part of ecological (Olson et al. 2001) and geographic changes (Linck et al. 2020). Both shape the landscape within which selection, adaptation, and extinction take place (Wu et al. 2022), including the historical geography or paleogeography at the time of divergence and speciation (Scotese 2016; Müller et al. 2018). This includes the factorization of well-established phenomena that preceded contemporary geography such as continental drift and known major periods of glaciation (Fig. 1), which can only be factored into the evolutionary history of a lineage or species if the time periods for diversification are known. For example, some species considered to be conspecific based on continuous distribution maps may have experienced historical barriers to gene flow that drove speciation millions of years ago. Conversely, some habitats may have previously been unsuitable for a species and have only become suitable for recolonization more recently (Olson et al. 2008; Le et al. 2022).

In studying evolutionary rates, accurate estimates of diversification rates within lineages are also dependent on comparing the temporal range within which the species, and subspecies, of a particular lineage are formed (Jetz et al. 2012). This is pertinent when comparing evolutionary rates between lineages to determine diversification rates, e.g., passerine bird species and non-passerines (Jetz et al. 2012); identifying lineages of interest in the study of speciation due to higher diversification rates (O'Connell et al. 2019); or in studying complex historical speciation events (Lamichhaney et al. 2018). It is, therefore, crucial that studies on evolutionary processes are contextualized within relevant time frames.

Over the past few decades, a plethora of molecular studies have been published using variable methods from fossil-calibrated Bayesian inference (Rannala and Yang 2003; Kumar and Hedges 2016) to comparable relative time approaches (Yang and Yoder 2003; Tamura et al. 2012) to establish the timeline for the emergence and diversification. This includes both living and extinct species within several lineages of mammals (Nyakatura and Bininda-Emonds 2012; Springer et al. 2018), reptiles (Tucker et al. 2017), and birds (Barker et al. 2015; Prum et al. 2015). These studies have greatly advanced our understanding of evolutionary processes within the context of environmental changes and the time constraints that they occur in (Scholl and Wiens 2016). They have also helped clarify many of the questions we have with regard to the taxonomy and phylogeny of species, which have frequently been at odds with each other (Sangster 2014; Springer et al. 2018). More specifically, time trees and gene trees do not always have identical topology as gene trees represent the variable substitution and mutation rates in lineages (Lanfear et al. 2010) which can obscure true species divergence times if not calibrated with more accurate divergence times (Tiley et al. 2020).

The result is a compendium of thousands of studies that has culminated in a central TimeTree resource (Hedges et al. 2006) that collects and compiles divergence time estimates and time trees from published and peer-reviewed studies. From this resource, estimates of divergence times, related timelines, and time trees are available online (Kumar et al. 2017). This also includes the ability to apply the use of TimeTree in MEGA (Mello 2018) and a mobile phone app (Kumar and Hedges 2011). Collectively this provides access to divergence time estimates for use in calibrating phylogenetic trees according to time, comparing clades in phylogenetic trees to know clades of shared common ancestry, as well as comparing genetic distance between species to their temporal or evolutionary distance. There is, however, still limited functionality in retrieving divergence times from the resource when dealing with species lists rather than individual pairs of species for downstream analyses or incorporation in molecular studies. As evolutionary studies frequently focus on multiple species, and even multiple lineages, at a time, this presents a significant barrier towards streamlining the integration of divergence time data into larger studies.

Previous attempts at automation (Krah 2015) to facilitate batch retrieval provided limited utilities and were poorly maintained, resulting in the removal of the package from the CRAN repository; although archived versions may still be available. The TimeTree resource has continued to develop and expand with the latest release of TimeTree 5, and the need for such capabilities is eminent in the ever-expanding field of evolutionary biology. While an application programming interface (API) has recently been created (Kumar et al. 2022), the use of APIs for the interaction with a database is not very biologist-friendly and requires additional computational skills. To bridge the current need, we have endeavored to create an easily accessible and freely available algorithm to retrieve relevant data on evolutionary histories from the TimeTree site for the seamless integration of divergence time data in molecular studies. In this paper we report on the PAReTT (Python-Automated Retrieval of TimeTree data), a menu-driven and user-friendly PYTHON package to automate interaction with the TimeTree resource for retrieving batch data with lists of species. We further provide several case examples to illustrate data retrieval and incorporation of data into scientific studies. The package is freely available on GitHub or as a stand-alone Windows executable.

## Methods

### Implementation

PAReTT (version 1.0.2) was scripted in the Spyder 5 IDE in pure PYTHON and is compatible with versions 3.6 and upward. A full list of dependencies is provided on the GitHub wiki, along with details for download and installation. This includes the use of several well-established PYTHON-based libraries such as NumPy (version 1.20.1) and pandas (version 1.2.4) for ease of input and high-order molding of data structures with relative ease (McKinney 2010; Harris et al. 2020), as well as Bio (version 1.3.9) for handling trees in the Newick format. PAReTT further uses the headless browser functionality implemented in Splinter (version 0.17.0) with the Selenium (version 4.1.5) extensions for the Firefox browser to submit user-specified web data to the TimeTree website (www.timetree.org) and retrieve the relevant results. Results are printed in real time to the shell, while list results are first stored to a ‘dataframe’ object which is written to user-specified output file. Some functionality had also been provided to validate data for any errors that may have occurred and preview basic tree files. Furthermore, the Magallon–Sanderson equation (Magallón and Sanderson 2001) was used to provide an option to calculate the diversification rate (*r*) of a lineage, genus, or species complex for which the number of extant species and divergence times are known. The *extinction ratio* (ε) is the fraction of the *extinction rate* (μ) divided by *speciation rate* (λ) according to the following equation:

1 $${\text{Extinction}}\,{\text{ratio }}\left( {\upvarepsilon } \right) = {\raise0.7ex\hbox{$\mu $} \!\mathord{\left/ {\vphantom {\mu \lambda }}\right.\kern-0pt} \!\lower0.7ex\hbox{$\lambda $}}.$$

The extinction rate represents the number of species formed that goes extinct in a given time period while the speciation rate represents the number of species that form within a given time period independent of survival outcomes.

When the *extinction ratio* is not known, the equation, with crown age, is given by (*n* is *total known species* and Δ*t* is *divergence time*):

2 $${\text{Diversification}}\,{\text{rate}} \left( r \right) = {\raise0.7ex\hbox{${\log n - \log 2}$} \!\mathord{\left/ {\vphantom {{\log n - \log 2} {\Delta t}}}\right.\kern-0pt} \!\lower0.7ex\hbox{${\Delta t}$}}.$$

When the extinction rate is known, the equation, with crown age, is given by

3 $$r = \frac{1}{\Delta t} \times \left( {\log \left( {\frac{{n\left( {1 - \varepsilon^{2} } \right)}}{2} + 2\varepsilon + \frac{{\left( {1 - \varepsilon } \right)}}{2} \times \sqrt[{}]{{n\left( {n\varepsilon^{2} - 8\varepsilon + 2n\varepsilon + n} \right)}}} \right) - \log 2} \right).$$

The diversification rate calculations make use of the math modules that form part of the standard PYTHON releases. The PAReTT script was benchmarked to test for time and memory consumption of individual functions using memory-profiler version 0.16.0.

### Input and Output File Formats

PAReTT uses two primary forms of input that users specify when the user is prompted to provide the name of the input file by the interactive menus. The first is a basic text file, indicated as ‘.txt,’ while the second is a standard comma-separated value (CSV) file, indicated as ‘.csv.’ While it is preferred to specify the full name of the file (e.g., ‘Species.txt’), PAReTT was scripted with checkpoints to ensure the proper format of input files, even when only a name is given, and should read the input files as long as they are in the current working directory. If the files are not stored in the current working directory, then the full file path name is preferred. The specific requirements for the content of each input file are detailed under usage in Section ‘Usage.’

Output files differ based on the specific data being retrieved. For example, timelines are retrieved as figures in the JPEG format while time trees, for both complete taxa as well as lists of species, are retrieved in the Newick format. Any species in the provided list for which the divergence time could not be resolved in the tree or were substituted by a similar species are stored as a table for review. Trees can be visualized and edited by most phylogenetic software programs including FigTree (Rambaut 2017) and MEGA (Kumar et al. 2018); however, the basic topology of these trees can also be viewed directly in PAReTT. Divergence time data, based on the median time from all studies from which divergence times estimates are derived, are printed on screen for pairs (only two species). Divergence times for lists of species will iterate through every possible combination of pairs in the lists and store data in ‘dataframe’ objects. These objects can then be stored in the output file as a vectorized three column matrix in a comma-separated value file. Output files are stored in the active working directory.

### Usage

**Fig. 2:**
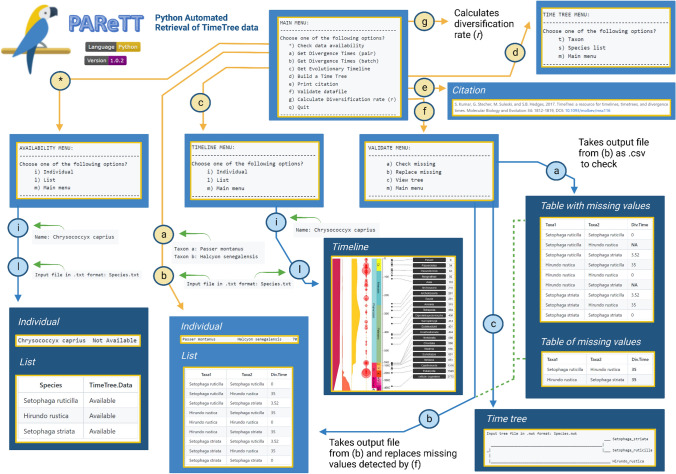
Graphical summary of menu options and submenus for PAReTT. The main menu options are: *, to verify the availability of data; **a** or **b** to resolve divergence times (pair or batch); **c** retrieve a timeline; **d** retrieve a time tree; **e**, print the TimeTree citation; **f** validate data; or **q** to exit. The data availability, timeline, and time tree options bring up a submenu, indicated by yellow arrows, to retrieve information for an individual species/taxon or a list. Output generated for lists are exported as a table (CSV), images (JPEG), or trees (Newick). The validation option brings up the choice of finding or replacing missing values for divergence times data (options a or b) as well as to view the tree topology (option c), for output files. (Image created in BioRender.com)

The main menu presents the initial options to verify the availability of data for a species, determine divergence times, a timeline, a time tree, validate the data, print the citation for TimeTree, or calculate divergence times as summarized in Fig. 2. The data availability option brings up a submenu to specify if availability should be verified for a single species or species list. When checking a single species, the availability is printed to the screen as the species name followed by either ‘Available’ or ‘Not Available.’ This function was designed to ensure data availability prior to further data retrieval steps. For a list of species, the species name and availability are printed on screen and stored to export results in a CSV file.

The timeline and time tree options will bring up a similar submenu that provides the option to retrieve information for an individual species, individual taxon, or a list of species. As before, individual species will result in a single output. The timeline retrieval function will retrieve an image in the JPEG format that illustrates the evolutionary history for the species highlighting the major time points where a kingdom, order, class, genus, and species first emerged. As an example, a timeline was retrieved for the Lazuli bunting, *Passerina amoena* [Say, 1823] in Example A. Input given as a list will retrieve individual images for each species in the list. For this example, we used the knowledge of divergence times from the timeline to compliment the timeline data with other relevant information such as the paleogeography (Sampson et al. 2010) and range data from *Birds of the World* (Greene et al. 2020).

The time tree option has similar functionality except that input can either be a larger taxonomic group, such as family or genus containing several congenic species, or a list of species as this output provides a time-calibrated tree displaying both the interrelatedness of species as well as the timescale along which the branches diverged. In Example B, two individual time trees were generated for the Nearctic thrushes (genus: *Catharus*) and Australasian fairywrens (genus: *Malurus*), respectively. The individual diversification rate (*r*) was calculated for each genus in PAReTT with the Magallon–Sanderson equation (Magallón and Sanderson 2001). More detailed speciation, extinction, and diversification rates (Etienne and Apol 2009) were computed with PyRate version 3.1.1 to further illustrate the use of diversification rates in evolutionary studies (Silvestro et al. 2014).

The Divergence time submenu (Example C) provides the option to check for the divergence time between two species e.g., the Neotropical Swainson’s thrush, *Catharus ustulatus* [Nuttall, 1840], and the Australasian Superb fairywren, *Malurus cyaneus* [Ellis, 1782]*.* The result will be printed to the screen as the name of ‘Taxon a’ and ‘Taxon b’ followed by the mean divergence time in millions of years ago (MYA) e.g., 35 MYA. The list option takes a text file with a list of species names and iterates through the list to determine the divergence time between each species in the list (Supplementary Fig. 1). The results are stored in a ‘dataframe’ object which can be exported as a CSV file in the form of a three column vectorized matrix with the names for ‘Taxa 1’ in the first column, the names of ‘Taxa 2’ in the second column, and the divergence times between them in the third column. Additional functionality is provided in the main menu to validate data. This option can be used to check output files for missing values, as well as retrieve and replace such values, in case a server error occurred. The table generated for species lists, in the form of a three column vectorized matrix, can then be converted to a full matrix to use divergence times as a measure of separation or differentness in Mantel tests (Mantel 1967; Carr 2021) as applied to landscape or spatial genetics. This was done to compare the distribution of genetic distance, as measured by fixation index (F_ST_) calculated with POPGENE version 1.32 (Yeh et al. 1997), for two circadian clock gene polymorphisms as they relate to evolutionary histories among forty bird species (Le Clercq et al. 2023).

## Results

### Example A—Timelines and Paleogeography

**Fig. 3:**
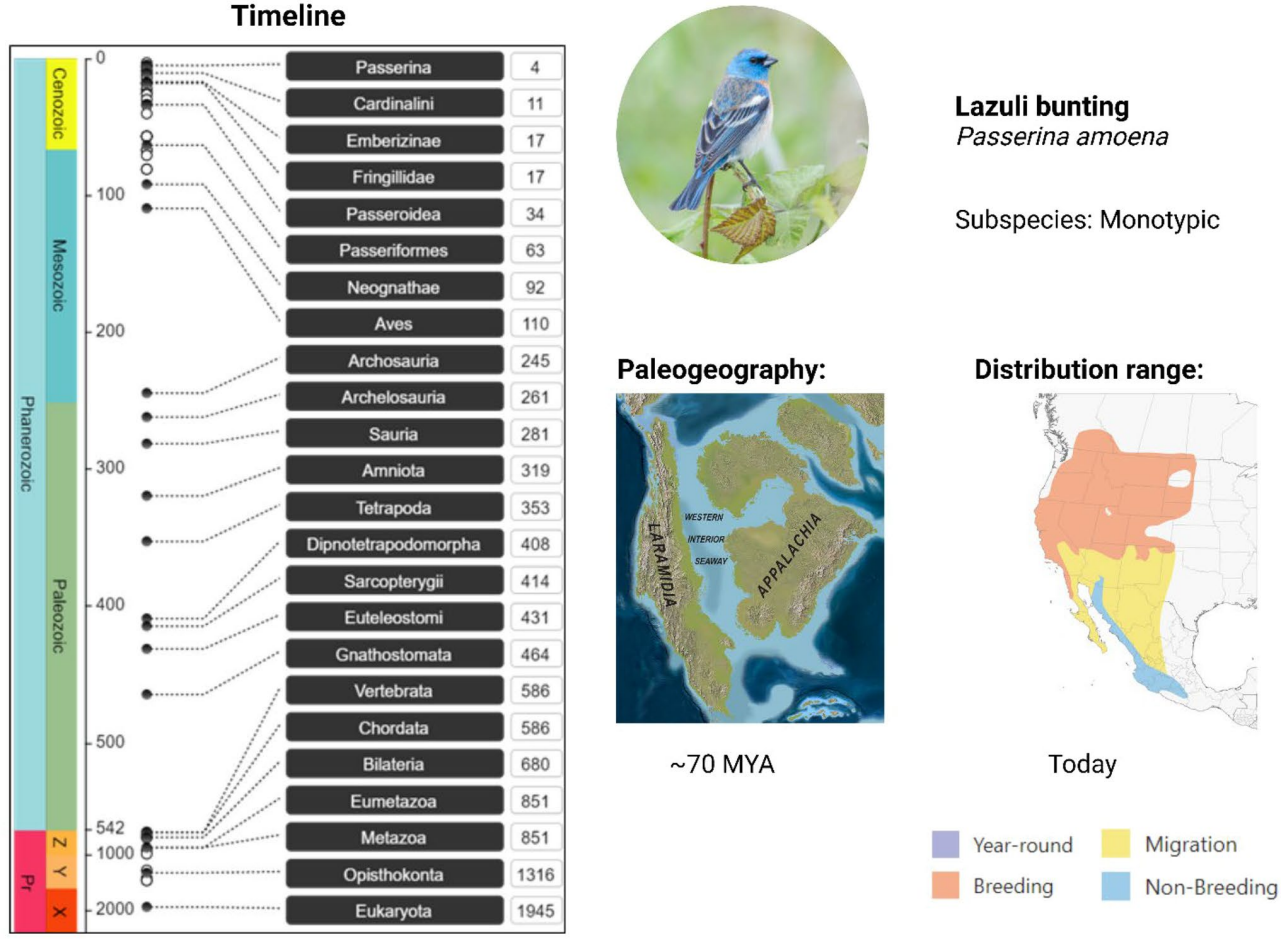
Example of an evolutionary timeline (left) retrieved using PAReTT for the Lazuli bunting. The left panel of the timeline indicates the major geologic timescale of the past 2000 MYA including the prevailing Phanerozoic Eon as well as the subdivisions by era Paleozoic which lasted until approximately 250 MYA when the Mesozoic started which lasted until 65 MYA. As is illustrated, this period was marked by the emergence of the first birds in the class Aves approximately 110 MYA. The species currently recognized in the family Cardinalidae emerged in the Cenozoic era approximately 11 MYA, with most species of the genus *Passerina* first emerging 4 MYA. On the right and in the middle, the paleogeography of the North American continent during this period is illustrated, clearly showing the separation of the Western and Eastern parts by the Western interior seaway (Sampson et al. 2010). Next to this is the contemporary range map for the species showing their breeding and wintering grounds that still encompass most of the Western half of the continent (Greene et al. 2020). (Image created in BioRender.com)

The timeline (Fig. 3) was retrieved for the Neotropical bird, the Lazuli bunting. The left panel indicates the major geologic timescales in Eons and Eras while the right indicates the main divergence times when specific taxa emerged. This includes the current Phanerozoic Eon as well as the subdivisions from the Paleozoic Era, which lasted until approximately 250 MYA, and the Mesozoic which lasted until 65 MYA illustrated in the first panel of Fig. 1. This period was marked by the emergence of several major lineages including the first birds (class: Aves) approximately 110 MYA as well as the taxonomic order *Passeriformes,* which includes most of the songbirds, approximately 63 MYA. The specific species, a bunting in the family Cardinalidae, emerged from a common ancestor shared with other cardinals in the Cenozoic approximately 11 MYA, while species of the genus *Passerina* first emerged approximately 4 MYA and subsequently diverged to form species. The range of the Lazuli bunting is located on the Western half of North America (Greene et al. 2020) which was physically separated from the Eastern half by the North American inland sea; the modern continent only formed after the Upper Cretaceous period around the Paleocene (Fig. 1).

### Example B—Time Trees and Diversification Rates

**Fig. 4:**
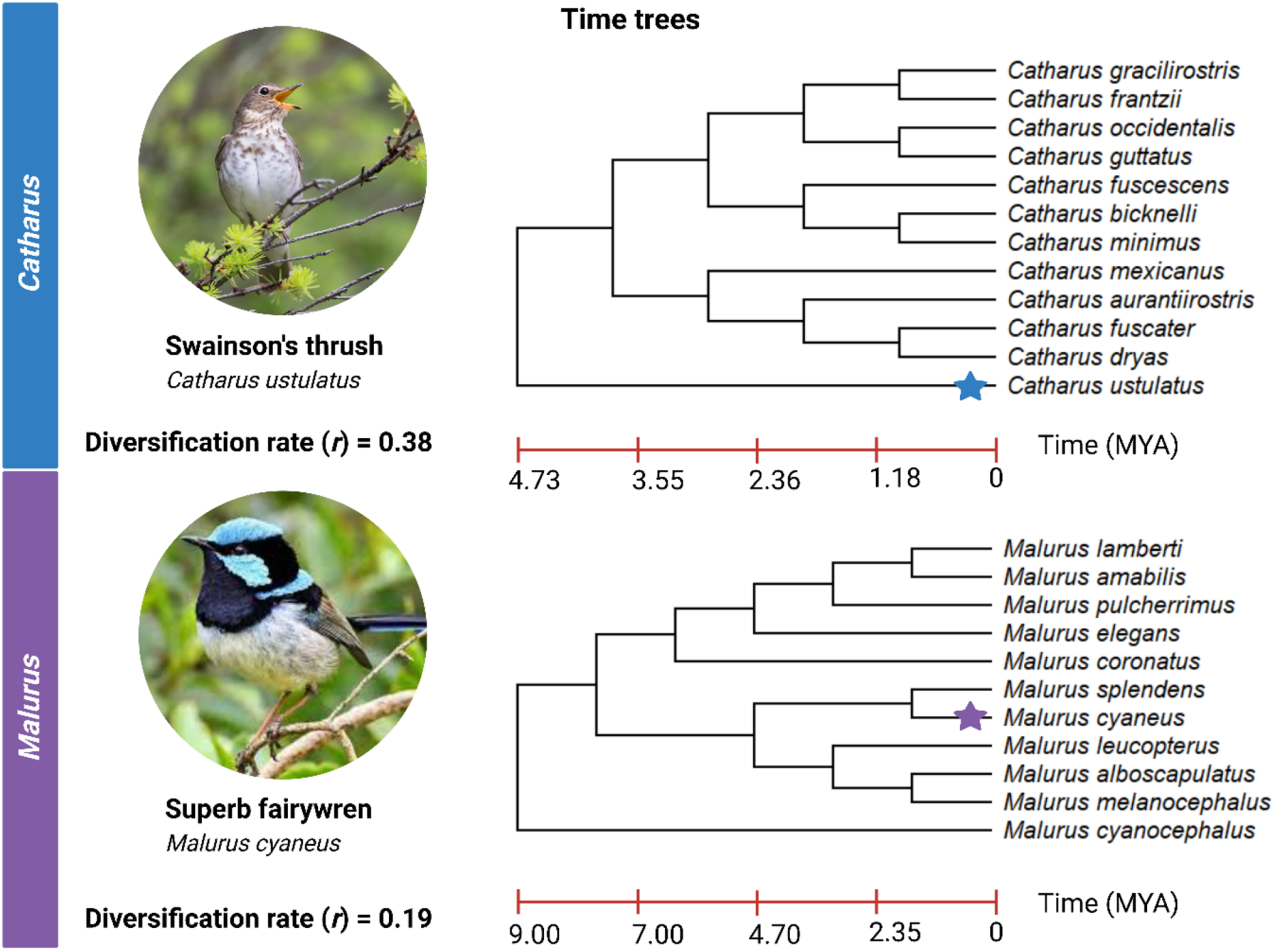
Comparison of time trees retrieved for two genera using PAReTT, one for several species in the *Catharus* genus of Neotropical thrushes and another of species in the *Malurus* genus of Australasian fairywrens. The *Catharus* genus has 12 species, including Swainson’s thrush, which diverged over a period of 4.74 MYA with a diversification rate of 0.38. The *Malurus* genus has 11 species, including the Superb fairywren, which diverged over a period of 9 MYA with a diversification rate of 0.19. This illustrates how two lineages may have similar evolutionary processes happening but have different diversification rates due to the difference in time scales. (Image created in BioRender.com)

**Fig. 5:**
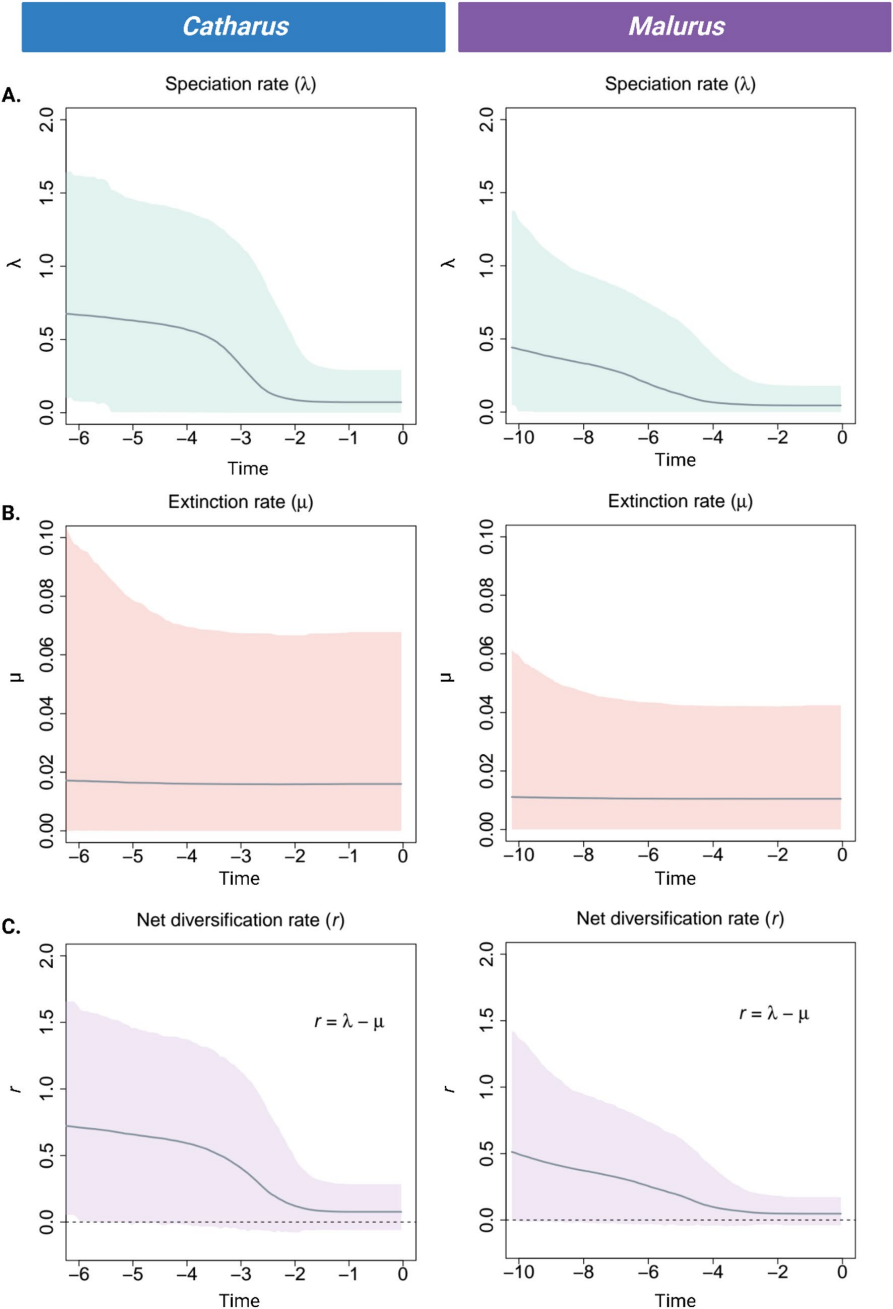
Plots from the PyRate analysis indicating the **A** speciation, **B** extinction, and **C** diversification rates over time for two genera of birds based on node age data from TimeTree. The left panel is the results for the *Catharus* thrush species complex while the right indicated the results for the *Malurus* fairywren species complex. Both genera followed similar patterns of speciation and diversification; however, the timescales over which these processes occurred are different and the *Catharus* species has slightly higher rates. As no extinct species could be included, the extinction rates (μ) are zero, resulting in highly similar speciation (λ) and diversification (*r*) plots. (Image created in BioRender.com)

Time trees (Fig. 4) were generated for two genera of birds that each contain several congenic species (11–12 species) but have different evolutionary histories in terms of the divergence times within their genus. The first represents the genus *Catharus* for which 12 species are included in the time tree. These species diverged over a period spanning approximately 4.73 million years and have an absolute diversification rate of 0.38 (*N* = 12). By comparison, the second tree for the genus *Malurus* includes a similar number of species; however, these species diverged over a period of approximately 9 million years and have a diversification rate (*r* = 0.19, *N* = 11) of nearly half the rate observed in *Catharus*. More detailed speciation and diversification rate estimates, for each species evolutionary timeline, are indicated in Fig. 5. Both genera displayed comparable trends in both speciation rate (λ) and diversification rate (*r*); however, these processes happened on different timescales, since approximately 10 MYA for *Malurus* species and 6 MYA for *Catharus* species. Overall, the rates for *Catharus* species were slightly higher. As extinct species for these lineages are unknown, the estimates for extinction rates (μ) were close to zero, resulting in highly similar speciation and diversification plots.

### Example C—Divergence Times and Genetic Distance

**Fig. 6:**
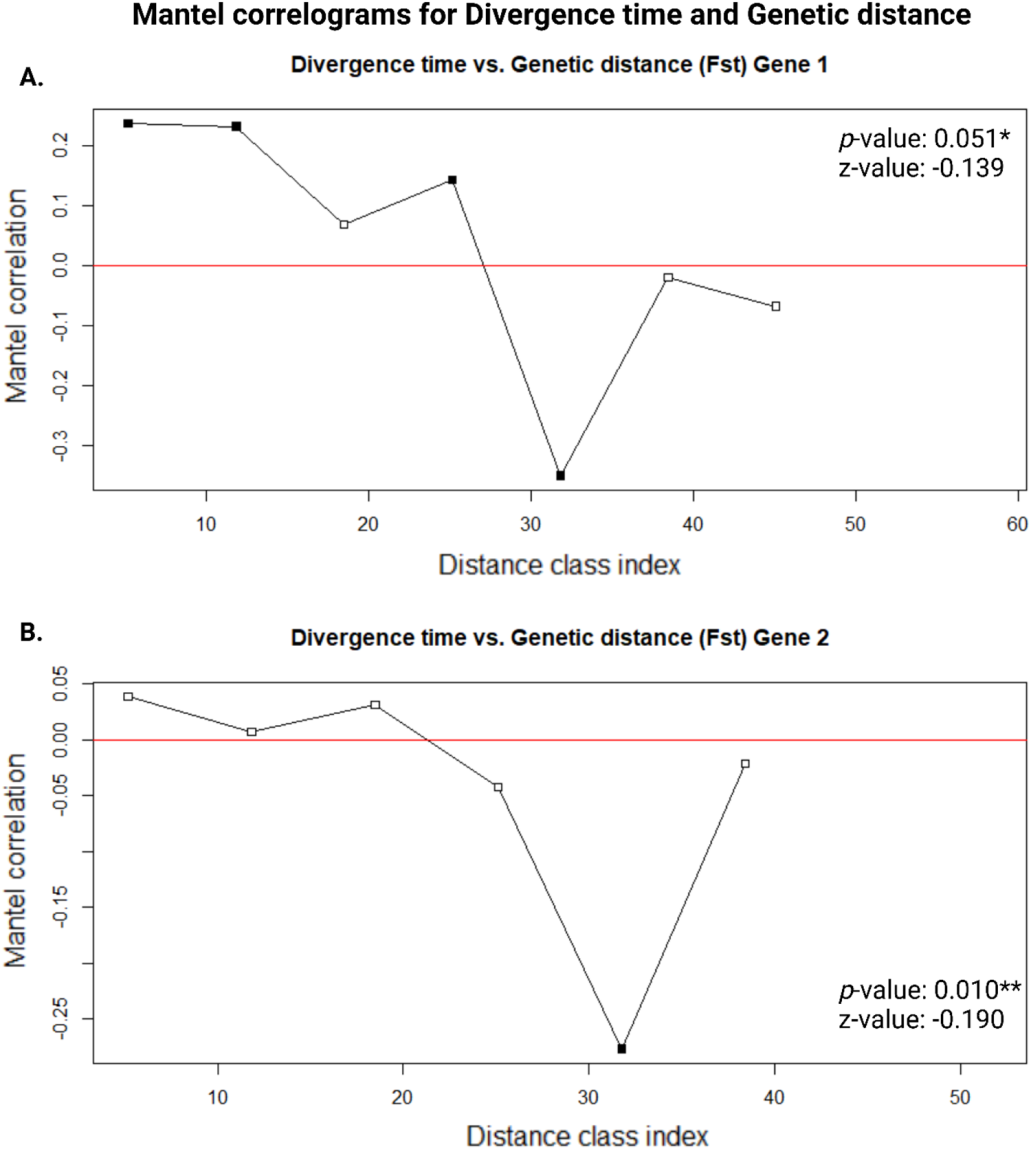
Mantel correlograms from the comparison of distance as measured by divergence times to genetic distance for gene 1 (**A**) and gene 2 (**B**). Divergence times were retrieved for forty bird species using PAReTT while genetic distance was measured by computing the fixation index (F_ST_) for two individual genes. For both genes, a significant correlation was found between the genetic distance and divergence times where increases in genetic distance corresponded to older divergence times. (*p-value < 0.1, **p-value < 0.05)

As an example, the divergence times retrieved for four species are given in Table 1: Lazuli bunting, Malachite kingfisher (*Corythornis cristatus* [Pallas, 1764]), Amethyst sunbird (*Chalcomitra amethystine* [Shaw, 1812]), and Emerald cuckoo (*Chrysococcyx cupreus* [Shaw, 1792]). The results clearly illustrate the more recent divergence time (25.4–38.1 MYA) between Lazuli bunting and Amethyst sunbird, both members of the same order (order: Passeriformes), as well as the far-older divergence times (73.7–97.3 MYA) between birds from different orders such as the Malachite kingfisher (order: Coraciiformes) and Emerald cuckoo (order: Cuculiformes). Furthermore, PAReTT was used to assess data availability and retrieve divergence times between species for a list of forty bird species in a recent review and meta-analysis on clock genes as candidate genes in migration studies, published in *Biological Reviews* (Le Clercq et al. 2023). This study found a significant relationship between divergence times and the observed genetic distance as measured by fixation index (F_ST_), for allele data of two candidate genes, using Mantel tests for matrix comparison (Fig. 6). Thus, the alleles and their distributions potentially still reflect the ancestral state rather than contemporary changes due to selection. The results from the benchmarking tests, performed in tandem, showed an average time for submitting data and retrieving the result ranged from fifteen to thirty seconds and memory consumption remained low, ranging from < 100 to 400 Megabytes (Supplementary Table 1).

**Table 1:** Examples of divergence times retrieved using the batch retrieval option in PAReTT

Taxa 1	Taxa 2	Divergence time	CI*	Studies*
Lazuli bunting	Lazuli bunting	0.0 MYA	n. a	n. a
Lazuli bunting	Malachite kingfisher	70.0 MYA	64.5–80.0 MYA	16
Lazuli bunting	Amethyst sunbird	27.6 MYA	25.4–38.1 MYA	5
Lazuli bunting	Emerald cuckoo	80.0 MYA	73.7–97.3 MYA	3
Malachite kingfisher	Lazuli bunting	70.0 MYA	64.5–80.0 MYA	16
Malachite kingfisher	Malachite kingfisher	0.0 MYA	n. a	n. a
Malachite kingfisher	Amethyst sunbird	70.0 MYA	64.5–80.0 MYA	16
Malachite kingfisher	Emerald cuckoo	80.0 MYA	73.7–97.3 MYA	3
Amethyst sunbird	Lazuli bunting	27.6 MYA	25.4–38.1 MYA	5
Amethyst sunbird	Malachite kingfisher	70.0 MYA	64.5–80.0 MYA	16
Amethyst sunbird	Amethyst sunbird	0.0 MYA	n. a	n. a
Amethyst sunbird	Emerald cuckoo	80.0 MYA	73.7–97.3 MYA	3
Emerald cuckoo	Lazuli bunting	80.0 MYA	73.7–97.3 MYA	3
Emerald cuckoo	Malachite kingfisher	80.0 MYA	73.7–97.3 MYA	3
Emerald cuckoo	Amethyst sunbird	80.0 MYA	73.7–97.3 MYA	3
Emerald cuckoo	Emerald cuckoo	0.0 MYA	n. a	n. a

Data were retrieved for a list of four species to illustrinput file are detailed under usage in Sectate the vectorized matrix format from PAReTT indicating the two taxa that were compared as well as the divergence time as either the median or adjusted median value. The list included the Lazuli bunting, Malachite kingfisher, Amethyst sunbird, and Emerald cuckoo. Additional data, indicated with an asterisk (*), such as the confidence interval (CI) of the estimate as well as the number of studies used to derive the values, were retrieved from the TimeTree website (www.timetree.org)

## Discussion

In this paper, we used three examples to illustrate the types of data that can be retrieved using PAReTT as well as how these data can be incorporated into evolutionary studies. The first involved a detailed timeline of the evolutionary history for Lazuli buntings which enabled a direct comparison of divergence times to the relative paleogeography for their range. The observation that their modern range corresponds to geographic patterns during their divergence time is indicative of speciation happening during former geographic isolation due to the North American inland sea (Western interior seaway), without substantial range expansion after the continent formed. This may have contributed to the division between the Lazuli bunting and other Neotropical buntings such as the Painted bunting, *Passerina ciris* [Linnaeus, 1758], which has a range that is restricted to the South-East of North America (Lowther et al. 2020) and Indigo bunting, *Passerina cyanea* [Linnaeus, 1766], which has a North-Eastern range (Payne 2020), although the primary division between these lineages is only dated to approximately 4.48 MYA. Similar observations have been made in North American *Catharus* species of thrushes (Voelker et al. 2013).

This example clearly illustrates the added value of divergence data and accurate timelines in reconstructing and understanding evolutionary processes. While we did use paleogeography (Sampson et al. 2010) from the literature and range data from the *Birds of the World* species records (Greene et al. 2020), this should be achievable in most species. Paleogeography can be reconstructed for nearly any period using PALEOMAP (Scotese 2016), as illustrated in Fig. 1, while range data can be retrieved for most birds (BirdLife International and Handbook of the Birds of the World 2021) as well as many mammals, reptiles, amphibians, and fishes (International Union for Conservation of Nature (IUCN) 2022) for use in range mapping analyses.

The second example illustrated the retrieval of time trees at the species level for two specific genera for the purpose of visually representing the timescale during which speciation and diversification happen in different lineages. Diversification rate calculations within PAReTT are based on user input rather than tree input as accurate measures would require a completely sampled phylogeny which is often unavailable. The diversification rate, based solely on the species for which data were available on the TimeTree resource, differed substantially between the two genera: having both different node ages for each genus as well as differing in the number of species, while having similar phylogenetic tree topology. More detailed analyses of speciation and diversification over time showed higher initial speciation with an eventual decline, possibly indicating a rate shift, followed by a plateau of lower rates. This could be due to the analyses only evaluating speciation at the species level and not the subspecies level, as several species within both lineages are partitioned into subspecies. For both genera, the TimeTree data were fairly complete as only two additional *Catharus* species, and one additional *Malurus* species, are currently recognized. In this example speciation and diversification rate plots were nearly identical due to the lack of known extinct species. This is contrary to the results that could be obtained for genera such as *Acrocephalus* warblers that includes 42 species of which 6 insular forms, including the Pagan reed warbler (*Acrocephalus yamashinae* [Taka-Tsukasa, 1931]), are known to be extinct.

The study of speciation, extinction, and diversification rates are critical in identifying lineages that rapidly for evolutionary studies to elucidate key factors that confer evolutionary advantages or disadvantages (Jetz et al. 2012; O'Connell et al. 2019). In addition, identifying lineages with slower speciation are informative for conservation practices, considering such species may experience higher difficulty to adapt to changes in their environment and both speciation and diversification rates have been tied to range changes, a critical attribute used in assessing species viability (Castiglione et al. 2017).

The third example focused on the retrieval of divergence times between lists of species to enable the construction of a matrix where divergence time serves as a proxy for temporal evolutionary distance. This enabled the comparison of genetic distance to evolutionary distance using Mantel tests (Mantel 1967; Carr 2021). The significant correlation between the measures illustrated the high heritability of genotypes within lineages which, combined with and a lack of selection, portends the possibility of these genes reflecting ancestral states (Le Clercq et al. 2023). This illustrates the significance in studying divergence time data in relation to genetic data in molecular studies. The TimeTree resource does, however, still lack options to facilitate batch retrieval of divergence times for lists of species.

Several key differences still exist in the types and formats of data that can be retrieved using PAReTT versus the main website. When retrieving timelines, PAReTT will retrieve the full timeline including other relevant ecological attributes; if only the major epochs and period are required for the image, then this can be specified on the online version. When retrieving time trees, PAReTT will automatically retrieve trees at the species level while trees above the genus level can be retrieved for a family or order using the website. Additionally, as PAReTT is designed to retrieve data for downstream analyses, the time trees are exported as editable Newick format trees rather than images intended to be used as is. Special features of PAReTT include the ability to retrieve batch divergence times for a list of species as a vectorized matrix that is well suited to downstream data analyses of divergence times as matrix data, although further details such as confidence intervals and the number of included studies is best retrieved from the website as needed. PAReTT can also calculate diversification rates based on retrieved data to ascertain if different rates exist between lineages.

Here, we illustrated the use of a newly scripted PYTHON package called PAReTT that provides an added layer of functionality to the TimeTree resource that can enhance molecular and evolutionary studies. Future updates will include the ability to switch between scientific names and common names for species as well as the ability to calculate diversification rates for a table of multiple lineages.

## Supplementary Information

Below is the link to the electronic supplementary material.

Supplementary file1 (DOCX 33 KB)

Supplementary file2 (DOCX 180 KB)

## Data Availability

The custom Python script for PAReTT version 1.0.2 is available for download for installation from source code on GitHub (https://github.com/LSLeClercq/PAReTT), or as a Windows executable of version 1.0.1 from the Zenodo online depository (https://zenodo.org/record/6653321#.Y6MnNHZBw2w), and includes example files used for testing. Data used in this paper were deposited online (https://zenodo.org/record/7496660#.Y7A-aHZBw2x).
