# Supplementary material for: PAReTT: A Python Package for the Automated Retrieval and Management of Divergence Time Data from the TimeTree Resource for Downstream Analyses
Source: J Mol Evol. 2023 Apr 20;91(4):502–13. doi: 10.1007/s00239-023-10106-3 (PMC10277261; doi:10.1007/s00239-023-10106-3)
Supplement: Supplementary file 1 — Supplementary file1 (DOCX 33 KB) [file 239_2023_10106_MOESM1_ESM.docx]

Table 1: Table of memory consumption and speed profiling of individual functions as benchmarking.

Line # Mem usage Increment Occurrences Line Contents

=============================================================

22 99.684 MiB 295.957 MiB 3 @profile

23 def menu_choice():

24 """ Find out what the user wants to do next. """

25 99.684 MiB 0.000 MiB 3 print("MAIN MENU:")

26 99.684 MiB 0.000 MiB 3 print("----------------------------------------")

27 99.684 MiB 0.000 MiB 3 print("Choose one of the following options?")

28 99.684 MiB 0.000 MiB 3 print(" *) Check data availability")

29 99.684 MiB 0.000 MiB 3 print(" a) Get Divergence Times (pair)")

30 99.684 MiB 0.000 MiB 3 print(" b) Get Divergence Times (batch)")

31 99.684 MiB 0.000 MiB 3 print(" c) Get Evolutionary Timeline")

32 99.684 MiB 0.000 MiB 3 print(" d) Build a Time Tree")

33 99.684 MiB 0.000 MiB 3 print(" e) Print citation")

34 99.684 MiB 0.000 MiB 3 print(" f) Validate datafile")

35 99.684 MiB 0.000 MiB 3 print(" q) Quit")

36 99.684 MiB 0.000 MiB 3 print("----------------------------------------")

37 99.684 MiB -0.008 MiB 3 choice = input("Choice: ")

38 99.684 MiB 0.000 MiB 3 print()

39 99.684 MiB 0.000 MiB 3 if choice.lower() in ['*','a','b','c','d','e','f','q']:

40 99.684 MiB 0.000 MiB 3 return choice.lower()

41 else:

42 print(choice +"?")

43 print("Invalid option")

44 print()

45 Time.sleep(3)

46 return None

47 98.680 MiB 196.270 MiB 2 @profile

48 def check_avail_menu():

49 """Menu options for checking data availability function"""

50 98.680 MiB 0.000 MiB 2 print('AVAILABILITY MENU:')

51 98.680 MiB 0.000 MiB 2 print("----------------------------------------")

52 98.680 MiB 0.000 MiB 2 print("Choose one of the following options?")

53 98.680 MiB 0.000 MiB 2 print(" i) Individual")

54 98.680 MiB 0.000 MiB 2 print(" l) List")

55 98.680 MiB 0.000 MiB 2 print(" m) Main menu")

56 98.680 MiB 0.000 MiB 2 print("----------------------------------------")

57 98.680 MiB 0.000 MiB 2 choice = input("Choice: ")

58 98.680 MiB 0.000 MiB 2 print()

59 98.680 MiB 0.000 MiB 2 if choice.lower() in ['i','l','m']:

60 98.680 MiB 0.000 MiB 2 return choice.lower()

61 else:

62 print(choice +"?")

63 print("Invalid option")

64 Time.sleep(3)

65 return None

66 98.680 MiB 196.270 MiB 2 @profile

67 def check_available():

68 """Checks the time tree website to confirm if data is available for a species"""

69 98.680 MiB 196.270 MiB 2 choice = check_avail_menu()

70 98.680 MiB 0.000 MiB 2 if choice == "i":

71 97.590 MiB 0.000 MiB 1 name = input("Name: ")

72 98.574 MiB 0.984 MiB 1 with Browser('firefox', headless=True) as browser:

73 98.629 MiB 0.055 MiB 1 browser.visit('http://timetree.org')

74 98.723 MiB 0.094 MiB 1 browser.fill('timeline_taxon', name)

75 98.723 MiB 0.000 MiB 1 browser.find_by_id('timeline-search-button1').click()

76 98.723 MiB 0.000 MiB 1 check = browser.is_text_present('Evolutionary Timeline for',

wait_time=15)

77 98.723 MiB 0.000 MiB 1 if check is True:

78 98.723 MiB 0.000 MiB 1 value = 'Available'

79 98.723 MiB 0.000 MiB 1 time = tm.datetime.now()

80 98.723 MiB 0.000 MiB 1 print('{:<25}'.format(name), end="")

81 98.723 MiB 0.000 MiB 1 print('{:<25}'.format(value), end="")

82 98.723 MiB 0.000 MiB 1 print(time)

83 elif check is False:

84 value = 'Not Available'

85 time = tm.datetime.now()

86 print('{:<25}'.format(name), end="")

87 print('{:<25}'.format(value), end="")

88 print(time)

89 98.723 MiB 0.000 MiB 1 print("Done!")

90 98.738 MiB 0.016 MiB 1 print()

91 98.680 MiB 0.000 MiB 1 elif choice == 'l':

92 99.031 MiB 0.352 MiB 1 data = pd.DataFrame(columns = ['Species', 'TimeTree.Data'])

93 99.031 MiB 0.000 MiB 1 infile = input("Input file in .txt format: ")

94 99.031 MiB 0.000 MiB 1 if infile.endswith('.txt') is True:

95 99.031 MiB 0.000 MiB 1 pass

96 elif infile.endswith('.txt') is False:

97 infile = infile + '.txt'

98 99.031 MiB 0.000 MiB 1 try:

99 99.031 MiB 0.000 MiB 1 my_file = open(infile, "r")

100 except FileNotFoundError:

101 print("File not found")

102 return

103 else:

104 99.039 MiB 0.008 MiB 1 my_file = open(infile, "r")

105 99.039 MiB 0.000 MiB 1 content = my_file.read()

106 99.039 MiB 0.000 MiB 1 content_list = content.split("\n")

107 99.039 MiB 0.000 MiB 1 my_file.close()

108 99.039 MiB 0.000 MiB 1 converted_list =[]

109 99.039 MiB 0.000 MiB 4 for element in content_list:

110 99.039 MiB 0.000 MiB 3 converted_list.append(element.strip())

111 99.039 MiB 0.000 MiB 1 species = tuple(converted_list)

112 99.582 MiB 0.000 MiB 4 for i in range(0,len(species)):

113 99.582 MiB 0.000 MiB 3 name = species[i]

114 99.605 MiB 0.293 MiB 3 with Browser('firefox', headless=True) as browser:

115 99.582 MiB -0.047 MiB 3 browser.visit('http://timetree.org')

116 99.582 MiB 0.012 MiB 3 browser.fill('timeline_taxon', name)

117 99.582 MiB 0.000 MiB 3 browser.find_by_id('timeline-search-button1').click()

118 99.582 MiB 0.000 MiB 3 browser.is_text_present('Evolutionary Timeline for', wait_time=120)

119 99.582 MiB 0.000 MiB 3 check = browser.is_text_present('Evolutionary Timeline for',

wait_time=15)

120 99.582 MiB 0.000 MiB 3 if check is True:

121 99.582 MiB 0.000 MiB 3 value = 'Available'

122 99.582 MiB 0.000 MiB 3 time = tm.datetime.now()

123 99.582 MiB 0.000 MiB 3 print('{:<25}'.format(name), end="")

124 99.582 MiB 0.000 MiB 3 print('{:<25}'.format(value), end="")

125 99.582 MiB 0.000 MiB 3 print(time)

126 99.582 MiB 0.262 MiB 6 data = data.append({'Species': name, 'TimeTree.Data':

'Available'},

127 99.582 MiB 0.000 MiB 3 ignore_index=True)

128 elif check is False:

129 value = 'Not Available'

130 time = tm.datetime.now()

131 print('{:<25}'.format(name), end="")

132 print('{:<25}'.format(value), end="")

133 print(time)

134 print("Done!")

135 data = data.append({'Species': name, 'TimeTree.Data': 'Not

Available'},

136 ignore_index=True)

137 99.582 MiB 0.000 MiB 1 output_name = input("File name (.csv): ")

138 99.582 MiB 0.000 MiB 1 if output_name.endswith('.csv') is True:

139 99.582 MiB 0.000 MiB 1 pass

140 elif output_name.endswith('.csv') is False:

141 output_name = output_name + '.csv'

142 99.684 MiB 0.102 MiB 1 data.to_csv(output_name, index=False)

143 99.684 MiB 0.000 MiB 1 print("Done!")

144 99.684 MiB 0.000 MiB 1 print()

145 97.457 MiB 97.457 MiB 1 @profile

146 def div_times_sing():

147 """Get the times for a single pair specified as input"""

148 97.457 MiB 0.000 MiB 1 taxon_a = input("Taxon a: ")

149 97.484 MiB 0.027 MiB 1 taxon_b = input("Taxon b: ")

150 98.711 MiB 1.227 MiB 1 with Browser('firefox', headless=True) as browser:

151 98.715 MiB 0.004 MiB 1 browser.visit('http://timetree.org')

152 98.832 MiB 0.117 MiB 1 browser.fill('taxon_a', taxon_a)

153 98.832 MiB 0.000 MiB 1 browser.fill('taxon_b', taxon_b)

154 98.832 MiB 0.000 MiB 1 browser.find_by_id('pairwise-search-button1').click()

155 98.793 MiB -0.039 MiB 1 browser.is_text_present('Median Time', wait_time=320)

156 98.793 MiB 0.000 MiB 1 var_y = '#pairwise-results > text:nth-child(11)'

157 98.793 MiB 0.000 MiB 1 divtime = browser.find_by_css(var_y).first.value

158 98.793 MiB 0.000 MiB 1 divtime_2 = divtime.replace(' MYA', '')

159 98.793 MiB 0.000 MiB 1 time = tm.datetime.now()

160 98.793 MiB 0.000 MiB 1 print('{:<25}'.format(taxon_a), end="")

161 98.793 MiB 0.000 MiB 1 print('{:<25}'.format(taxon_b), end="")

162 98.793 MiB 0.000 MiB 1 print('{:<5}'.format(divtime_2), end="")

163 98.793 MiB 0.000 MiB 1 print(time)

164 98.809 MiB 0.016 MiB 1 print()

165 98.809 MiB 98.809 MiB 1 @profile

166 def div_times_batch():

167 """Get the divergence times for a list of species and stores it as a data-

168 frame which gets exported as a .csv with 3 columns"""

169 99.164 MiB 0.355 MiB 1 data = pd.DataFrame(columns = ['Taxa1', 'Taxa2', 'Div.Time'])

170 99.164 MiB 0.000 MiB 1 infile = input("Input file in .txt format: ")

171 99.164 MiB 0.000 MiB 1 if infile.endswith('.txt') is True:

172 99.164 MiB 0.000 MiB 1 pass

173 elif infile.endswith('.txt') is False:

174 infile = infile + '.txt'

175 99.164 MiB 0.000 MiB 1 try:

176 99.164 MiB 0.000 MiB 1 my_file = open(infile, "r")

177 except FileNotFoundError:

178 print("File not found")

179 return

180 else:

181 99.172 MiB 0.008 MiB 1 my_file = open(infile, "r")

182 99.172 MiB 0.000 MiB 1 content = my_file.read()

183 99.172 MiB 0.000 MiB 1 content_list = content.split("\n")

184 99.172 MiB 0.000 MiB 1 my_file.close()

185 99.172 MiB 0.000 MiB 1 converted_list =[]

186 99.172 MiB 0.000 MiB 4 for element in content_list:

187 99.172 MiB 0.000 MiB 3 converted_list.append(element.strip())

188 99.172 MiB 0.000 MiB 1 species = tuple(converted_list)

189 99.172 MiB 0.000 MiB 1 length = len(species)

190 99.625 MiB -0.004 MiB 4 for variable_x in range(0,length):

191 99.625 MiB -0.008 MiB 12 for i in range(0,length):

192 99.625 MiB -0.020 MiB 9 taxon_a = species[variable_x]

193 99.625 MiB -0.020 MiB 9 taxon_b = species[i]

194 99.625 MiB -0.020 MiB 9 if taxon_b == species[-1]:

195 99.625 MiB -0.008 MiB 3 if taxon_a == taxon_b:

196 99.625 MiB 0.000 MiB 1 time = tm.datetime.now()

197 99.625 MiB 0.000 MiB 1 print('{:<25}'.format(taxon_a), end="")

198 99.625 MiB 0.000 MiB 1 print('{:<25}'.format(taxon_b), end="")

199 99.625 MiB 0.000 MiB 1 print('{:<5}'.format('0'), end="")

200 99.625 MiB 0.000 MiB 1 print(time)

201 99.625 MiB 0.000 MiB 2 data = data.append({'Taxa1' : taxon_a, 'Taxa2' : taxon_b,

'Div.Time' : '0'},

202 99.625 MiB 0.000 MiB 1 ignore_index = True)

203 else:

204 99.574 MiB 0.059 MiB 2 with Browser('firefox', headless=True) as browser:

205 99.574 MiB 0.000 MiB 2 try:

206 99.578 MiB 0.004 MiB 2 browser.visit('http://timetree.org')

207 99.582 MiB 0.004 MiB 2 browser.fill('taxon_a', taxon_a)

208 99.582 MiB 0.000 MiB 2 browser.fill('taxon_b', taxon_b)

209 99.582 MiB 0.000 MiB 2 browser.find_by_id('pairwise-search-button1').click()

210 except:

211 Time.sleep(600)

212 browser.visit('http://timetree.org')

213 browser.fill('taxon_a', taxon_a)

214 browser.fill('taxon_b', taxon_b)

215 browser.find_by_id('pairwise-search-button1').click()

216 99.574 MiB -0.008 MiB 2 browser.is_text_present('Median Time', wait_time=320)

217 99.574 MiB 0.000 MiB 2 var_y = '#pairwise-results > text:nth-child(11)'

218 99.574 MiB 0.000 MiB 2 try:

219 99.578 MiB 0.008 MiB 2 divtime = browser.find_by_css(var_y).first.value

220 except:

221 divtime = 'NA MYA'

222 else:

223 99.578 MiB -0.004 MiB 2 divtime = browser.find_by_css(var_y).first.value

224 99.578 MiB -0.004 MiB 2 divtime_2 = divtime.replace(' MYA', '')

225 99.578 MiB -0.004 MiB 2 time = tm.datetime.now()

226 99.578 MiB -0.004 MiB 2 print('{:<25}'.format(taxon_a), end="")

227 99.578 MiB -0.004 MiB 2 print('{:<25}'.format(taxon_b), end="")

228 99.578 MiB -0.004 MiB 2 print('{:<5}'.format(divtime_2), end="")

229 99.578 MiB -0.004 MiB 2 print(time)

230 99.582 MiB -0.004 MiB 6 data = data.append({'Taxa1' : taxon_a, 'Taxa2' : taxon_b,

231 99.578 MiB -0.016 MiB 4 'Div.Time' : divtime_2}, ignore_index = True)

232 99.625 MiB -0.004 MiB 3 print(taxon_a,'DONE!')

233 else:

234 99.621 MiB -0.012 MiB 6 if taxon_a == taxon_b:

235 99.574 MiB -0.008 MiB 2 time = tm.datetime.now()

236 99.574 MiB 0.000 MiB 2 print('{:<25}'.format(taxon_a), end="")

237 99.574 MiB 0.000 MiB 2 print('{:<25}'.format(taxon_b), end="")

238 99.574 MiB 0.000 MiB 2 print('{:<5}'.format('0'), end="")

239 99.574 MiB 0.000 MiB 2 print(time)

240 99.574 MiB 0.180 MiB 4 data = data.append({'Taxa1' : taxon_a, 'Taxa2' : taxon_b,

'Div.Time' : '0'},

241 99.574 MiB 0.000 MiB 2 ignore_index = True)

242 else:

243 99.621 MiB 0.082 MiB 4 with Browser('firefox', headless=True) as browser:

244 99.621 MiB 0.000 MiB 4 try:

245 99.621 MiB 0.000 MiB 4 browser.visit('http://timetree.org')

246 99.621 MiB 0.098 MiB 4 browser.fill('taxon_a', taxon_a)

247 99.621 MiB 0.004 MiB 4 browser.fill('taxon_b', taxon_b)

248 99.621 MiB 0.004 MiB 4 browser.find_by_id('pairwise-search-button1').click()

249 except:

250 Time.sleep(600)

251 browser.visit('http://timetree.org')

252 browser.fill('taxon_a', taxon_a)

253 browser.fill('taxon_b', taxon_b)

254 browser.find_by_id('pairwise-search-button1').click()

255 99.621 MiB 0.004 MiB 4 browser.is_text_present('Median Time', wait_time=320)

256 99.621 MiB 0.000 MiB 4 var_y = '#pairwise-results > text:nth-child(11)'

257 99.621 MiB 0.000 MiB 4 try:

258 99.621 MiB 0.000 MiB 4 divtime = browser.find_by_css(var_y).first.value

259 except:

260 divtime = 'NA MYA'

261 else:

262 99.621 MiB 0.000 MiB 4 divtime = browser.find_by_css(var_y).first.value

263 99.621 MiB 0.000 MiB 4 divtime_2 = divtime.replace(' MYA', '')

264 99.621 MiB 0.000 MiB 4 time = tm.datetime.now()

265 99.621 MiB 0.000 MiB 4 print('{:<25}'.format(taxon_a), end="")

266 99.621 MiB 0.000 MiB 4 print('{:<25}'.format(taxon_b), end="")

267 99.621 MiB 0.000 MiB 4 print('{:<5}'.format(divtime_2), end="")

268 99.621 MiB 0.000 MiB 4 print(time)

269 99.625 MiB 0.004 MiB 12 data = data.append({'Taxa1' : taxon_a, 'Taxa2' : taxon_b,

270 99.621 MiB 0.000 MiB 8 'Div.Time' : divtime_2}, ignore_index = True)

271 99.625 MiB 0.000 MiB 1 if i == len(species):

272 print(taxon_a," DONE!")

273 99.645 MiB 0.020 MiB 1 data.replace(to_replace=[r"\\t|\\n|\\r", "\t|\n|\r"], value=["",""], regex=True,

inplace=True)

274 99.645 MiB 0.000 MiB 1 print()

275 99.832 MiB 0.188 MiB 1 print(data)

276 99.832 MiB 0.000 MiB 1 print()

277 99.832 MiB 0.000 MiB 1 output_name = input("File name (.csv): ")

278 99.832 MiB 0.000 MiB 1 if output_name.endswith('.csv') is True:

279 99.832 MiB 0.000 MiB 1 pass

280 elif output_name.endswith('.csv') is False:

281 output_name = output_name + '.csv'

282 99.961 MiB 0.129 MiB 1 data.to_csv(output_name, index=False)

283 99.961 MiB 0.000 MiB 1 print("Done!")

284 99.961 MiB 0.000 MiB 1 print()

285 98.828 MiB 196.559 MiB 2 @profile

286 def timeline_menu():

287 """Menu options for timeline function"""

288 98.828 MiB 0.000 MiB 2 print('TIMELINE MENU:')

289 98.828 MiB 0.000 MiB 2 print("----------------------------------------")

290 98.828 MiB 0.000 MiB 2 print("Choose one of the following options?")

291 98.828 MiB 0.000 MiB 2 print(" i) Individual")

292 98.828 MiB 0.000 MiB 2 print(" l) List")

293 98.828 MiB 0.000 MiB 2 print(" m) Main menu")

294 98.828 MiB 0.000 MiB 2 print("----------------------------------------")

295 98.828 MiB 0.000 MiB 2 choice = input("Choice: ")

296 98.828 MiB 0.000 MiB 2 print()

297 98.828 MiB 0.000 MiB 2 if choice.lower() in ['i','l','m']:

298 98.828 MiB 0.000 MiB 2 return choice.lower()

299 else:

300 print(choice +"?")

301 print("Invalid option")

302 Time.sleep(3)

303 return None

304 98.828 MiB 196.559 MiB 2 @profile

305 def evol_timeline():

306 """Gets the evolutionary timeline for a species or taxa given a single

307 name or a list"""

308 98.828 MiB 196.559 MiB 2 choice = timeline_menu()

309 98.828 MiB 0.000 MiB 2 if choice == "i":

310 97.730 MiB 0.000 MiB 1 name = input("Name: ")

311 98.711 MiB 0.980 MiB 1 with Browser('firefox', headless=True) as browser:

312 98.762 MiB 0.051 MiB 1 browser.visit('http://timetree.org')

313 98.859 MiB 0.098 MiB 1 browser.fill('timeline_taxon', name)

314 98.859 MiB 0.000 MiB 1 browser.find_by_id('timeline-search-button1').click()

315 98.863 MiB 0.004 MiB 1 browser.is_text_present('Evolutionary Timeline for', wait_time=15)

316 98.863 MiB 0.000 MiB 1 browser.find_by_id('timeline-timeline-svg-export-btn').click()

317 98.863 MiB 98.863 MiB 1 downloads = get_download_path()

318 98.863 MiB 0.000 MiB 1 current = os.getcwd()

319 98.863 MiB 0.000 MiB 1 file = name + '_' + 'timeline.jpg'

320 98.863 MiB 0.000 MiB 1 indir = str(downloads) + '\\' + file

321 98.863 MiB 0.000 MiB 1 outdir = str(current) + '\\' + file

322 98.871 MiB 0.008 MiB 1 shutil.move(indir, outdir)

323 98.871 MiB 0.000 MiB 1 print("Done!")

324 98.887 MiB 0.016 MiB 1 print()

325 98.828 MiB 0.000 MiB 1 elif choice == 'l':

326 98.828 MiB 0.000 MiB 1 infile = input("Input file in .txt format: ")

327 98.828 MiB 0.000 MiB 1 if infile.endswith('.txt') is True:

328 98.828 MiB 0.000 MiB 1 pass

329 elif infile.endswith('.txt') is False:

330 infile = infile + '.txt'

331 98.828 MiB 0.000 MiB 1 try:

332 98.828 MiB 0.000 MiB 1 my_file = open(infile, "r")

333 except FileNotFoundError:

334 print("File not found")

335 return

336 else:

337 98.836 MiB 0.008 MiB 1 my_file = open(infile, "r")

338 98.836 MiB 0.000 MiB 1 content = my_file.read()

339 98.836 MiB 0.000 MiB 1 content_list = content.split("\n")

340 98.836 MiB 0.000 MiB 1 my_file.close()

341 98.836 MiB 0.000 MiB 1 converted_list =[]

342 98.836 MiB 0.000 MiB 4 for element in content_list:

343 98.836 MiB 0.000 MiB 3 converted_list.append(element.strip())

344 98.836 MiB 0.000 MiB 1 species = tuple(converted_list)

345 99.297 MiB 0.000 MiB 4 for i in range(0,len(species)):

346 99.234 MiB 0.000 MiB 3 name = species[i]

347 99.297 MiB 0.305 MiB 3 with Browser('firefox', headless=True) as browser:

348 99.297 MiB 0.008 MiB 3 browser.visit('http://timetree.org')

349 99.297 MiB 0.105 MiB 3 browser.fill('timeline_taxon', name)

350 99.297 MiB 0.062 MiB 3 browser.find_by_id('timeline-search-button1').click()

351 99.297 MiB 0.004 MiB 3 browser.is_text_present('Evolutionary Timeline for', wait_time=120)

352 99.297 MiB -0.023 MiB 3 browser.find_by_id('timeline-timeline-svg-export-btn').click()

353 99.297 MiB 0.000 MiB 3 time = tm.datetime.now()

354 99.297 MiB 0.000 MiB 3 print('{:<25}'.format(name), end="")

355 99.297 MiB 0.000 MiB 3 print(time)

356 99.297 MiB 297.707 MiB 3 downloads = get_download_path()

357 99.297 MiB 0.000 MiB 3 current = os.getcwd()

358 99.297 MiB 0.000 MiB 3 file = name + '_' + 'timeline.jpg'

359 99.297 MiB 0.000 MiB 3 indir = str(downloads) + '\\' + file

360 99.297 MiB 0.000 MiB 3 outdir = str(current) + '\\' + file

361 99.297 MiB 0.000 MiB 3 shutil.move(indir, outdir)

362 99.297 MiB 0.000 MiB 1 print("Done!")

363 99.297 MiB 0.000 MiB 1 print()

364 elif choice == 'm':

365 main_loop()

366 98.832 MiB 196.523 MiB 2 @profile

367 def time_tree_menu():

368 """Menu options for time tree function"""

369 98.832 MiB 0.000 MiB 2 print('TIME TREE MENU:')

370 98.832 MiB 0.000 MiB 2 print("----------------------------------------")

371 98.832 MiB 0.000 MiB 2 print("Choose one of the following options?")

372 98.832 MiB 0.000 MiB 2 print(" t) Taxon")

373 98.832 MiB 0.000 MiB 2 print(" s) Species list")

374 98.832 MiB 0.000 MiB 2 print(" m) Main menu")

375 98.832 MiB 0.000 MiB 2 print("----------------------------------------")

376 98.832 MiB 0.000 MiB 2 choice = input("Choice: ")

377 98.832 MiB 0.000 MiB 2 if choice.lower() in ['t','s','m']:

378 98.832 MiB 0.000 MiB 2 return choice.lower()

379 else:

380 print(choice +"?")

381 print("Invalid option")

382 Time.sleep(3)

383 return None

384 98.832 MiB 196.523 MiB 2 @profile

385 def time_tree():

386 """Takes a species list as input and returns a time tree in one of the specified

387 formats"""

388 98.832 MiB 196.523 MiB 2 choice = time_tree_menu()

389 98.832 MiB 0.000 MiB 2 if choice == 't':

390 97.691 MiB 0.000 MiB 1 name = input("Name: ")

391 98.734 MiB 1.043 MiB 1 with Browser('firefox', headless=True) as browser:

392 98.805 MiB 0.070 MiB 1 browser.visit('http://timetree.org')

393 98.852 MiB 0.047 MiB 1 browser.fill('timetree_taxon', name)

394 98.855 MiB 0.004 MiB 1 browser.find_by_id('timetree-search-button1').click()

395 98.859 MiB 0.004 MiB 1 browser.find_by_id('generate-download-button').click()

396 98.859 MiB 98.859 MiB 1 downloads = get_download_path()

397 98.859 MiB 0.000 MiB 1 current = os.getcwd()

398 98.859 MiB 0.000 MiB 1 file = name + '_' + 'species.nwk'

399 98.859 MiB 0.000 MiB 1 indir = str(downloads) + '\\' + file

400 98.859 MiB 0.000 MiB 1 outdir = str(current) + '\\' + file

401 98.867 MiB 0.008 MiB 1 shutil.move(indir, outdir)

402 98.883 MiB 0.016 MiB 1 print("Done!")

403 98.883 MiB 0.000 MiB 1 print()

404 98.832 MiB 0.000 MiB 1 elif choice == 's':

405 98.832 MiB 0.000 MiB 1 infile_name = input("Input file in .txt format: ")

406 98.832 MiB 0.000 MiB 1 if infile_name.endswith('.txt') is True:

407 98.832 MiB 0.000 MiB 1 pass

408 elif infile_name.endswith('.txt') is False:

409 infile_name = infile_name + '.txt'

410 98.832 MiB 0.000 MiB 1 try:

411 98.840 MiB 0.008 MiB 1 open(infile_name, "r")

412 except FileNotFoundError:

413 print("File not found")

414 return

415 98.840 MiB 0.000 MiB 1 if os.path.isfile(infile_name) is True:

416 98.840 MiB 0.000 MiB 1 pass

417 elif os.path.isfile(infile_name) is False:

418 print("No such file!")

419 return

420 98.840 MiB 0.000 MiB 1 path = os.getcwd()

421 98.840 MiB 0.000 MiB 1 infile_path = path + "\\" + infile_name

422 98.840 MiB 0.000 MiB 1 print(infile_path)

423 98.848 MiB 0.008 MiB 1 with Browser('firefox', headless=True) as browser:

424 98.840 MiB -0.008 MiB 1 browser.visit('http://timetree.org')

425 98.883 MiB 0.043 MiB 1 browser.attach_file("prunetree_upload", infile_path)

426 98.883 MiB 0.000 MiB 1 browser.find_by_id('prunetree-upload-button1').click()

427 98.895 MiB 0.012 MiB 1 browser.is_text_present('Geologic Timescale', wait_time=320)

428 98.895 MiB 0.000 MiB 1 browser.find_by_id('prunetree-msg-btn').click()

429 98.895 MiB 0.000 MiB 1 try:

430 98.902 MiB 0.008 MiB 1 replaced = browser.find_by_id('unresolved-names').value

431 except BaseException:

432 pass

433 else:

434 98.902 MiB 0.000 MiB 1 replaced = browser.find_by_id('unresolved-names').value

435 98.902 MiB 0.000 MiB 1 file = open('Unresolved names.txt','w')

436 98.902 MiB 0.000 MiB 1 file.write(replaced)

437 98.902 MiB 0.000 MiB 1 file.close()

438 98.902 MiB 0.000 MiB 1 browser.find_by_id('prunetree-newick-export-btn').click()

439 98.902 MiB 98.902 MiB 1 downloads = get_download_path()

440 98.902 MiB 0.000 MiB 1 current = os.getcwd()

441 98.902 MiB 0.000 MiB 1 file = infile_name.replace('.txt', '.nwk')

442 98.902 MiB 0.000 MiB 1 indir = str(downloads) + '\\' + file

443 98.902 MiB 0.000 MiB 1 outdir = str(current) + '\\' + file

444 98.926 MiB 0.023 MiB 1 shutil.move(indir, outdir)

445 98.926 MiB 0.000 MiB 1 print("Done!")

446 98.926 MiB 0.000 MiB 1 print()

447 elif choice == 'm':

448 main_loop()

449 97.516 MiB 97.516 MiB 1 @profile

450 def citation():

451 """Prints the citation for the timetree resource"""

452 97.516 MiB 0.000 MiB 1 print()

453 97.516 MiB 0.000 MiB 1 print("CITE THE TIME TREE RESOURCE AS:")

454 97.516 MiB 0.000 MiB 2 print("S. Kumar, G. Stecher, M. Suleski, and S.B. Hedges, 2017.",

455 97.516 MiB 0.000 MiB 1 " TimeTree: a resource for timelines, timetrees, and divergence times.",

456 97.516 MiB 0.000 MiB 1 " Molecular Biology and Evolution 34: 1812-1819,",

457 97.516 MiB 0.000 MiB 1 " DOI: 10.1093/molbev/msx116")

458 97.516 MiB 0.000 MiB 1 print()

459 100.078 MiB 297.543 MiB 3 @profile

460 def validate_menu():

461 """Menu options for validate function"""

462 100.078 MiB 0.000 MiB 3 print("VALIDATE MENU:")

463 100.078 MiB 0.000 MiB 3 print("----------------------------------------")

464 100.078 MiB 0.000 MiB 3 print(" a) Check missing")

465 100.078 MiB 0.000 MiB 3 print(" b) Replace missing")

466 100.078 MiB 0.000 MiB 3 print(" c) View tree")

467 100.078 MiB 0.000 MiB 3 print(" m) Main menu")

468 100.078 MiB 0.000 MiB 3 print("----------------------------------------")

469 100.078 MiB -0.031 MiB 3 choice = input("Choice: ")

470 100.078 MiB 0.000 MiB 3 if choice.lower() in ['a','b','c','m']:

471 100.078 MiB 0.000 MiB 3 return choice.lower()

472 else:

473 print(choice +"?")

474 print("Invalid option")

475 Time.sleep(3)

476 return None

477 100.078 MiB 297.543 MiB 3 @profile

478 def validate():

479 """Checks output file for missing values"""

480 100.078 MiB 297.512 MiB 3 choice = validate_menu()

481 100.078 MiB 0.000 MiB 3 if choice == 'a':

482 97.488 MiB 0.000 MiB 1 infile = input("File to check (.csv): ")

483 97.488 MiB 0.000 MiB 1 if infile.endswith('.csv') is True:

484 97.488 MiB 0.000 MiB 1 pass

485 elif infile.endswith('.csv') is False:

486 infile = infile + '.csv'

487 97.922 MiB 0.434 MiB 1 data = pd.read_csv(infile)

488 97.922 MiB 0.000 MiB 1 length = len(data.index)

489 98.160 MiB 0.238 MiB 1 indices = data.loc[pd.isna(data).any(1), :].index

490 98.160 MiB 0.000 MiB 1 check = len(indices)

491 98.160 MiB 0.000 MiB 1 if check >= 1:

492 98.242 MiB 0.082 MiB 1 missing = pd.DataFrame(columns = ['Taxa1', 'Taxa2', 'Div.Time'])

493 99.910 MiB 0.000 MiB 10 for i in range (0, length):

494 99.910 MiB 0.066 MiB 9 if i in indices:

495 99.906 MiB 0.027 MiB 2 taxon_a = data.iloc[i]['Taxa1']

496 99.906 MiB 0.000 MiB 2 taxon_b = data.iloc[i]['Taxa2']

497 99.914 MiB 1.238 MiB 2 with Browser('firefox', headless=True) as browser:

498 99.914 MiB 0.000 MiB 2 try:

499 99.914 MiB 0.066 MiB 2 browser.visit('http://timetree.org')

500 99.914 MiB 0.047 MiB 2 browser.fill('taxon_a', taxon_a)

501 99.914 MiB 0.000 MiB 2 browser.fill('taxon_b', taxon_b)

502 99.914 MiB 0.000 MiB 2 browser.find_by_id('pairwise-search-button1').click()

503 except KeyboardInterrupt as error:

504 print('An exception occurred: {}'.format(error))

505 return

506 else:

507 99.922 MiB 0.008 MiB 2 browser.visit('http://timetree.org')

508 99.922 MiB 0.000 MiB 2 browser.fill('taxon_a', taxon_a)

509 99.922 MiB 0.000 MiB 2 browser.fill('taxon_b', taxon_b)

510 99.922 MiB 0.000 MiB 2 browser.find_by_id('pairwise-search-button1').click()

511 99.910 MiB -0.008 MiB 2 browser.is_text_present('Median Time', wait_time=320)

512 99.910 MiB 0.000 MiB 2 var_y = '#pairwise-results > text:nth-child(11)'

513 99.910 MiB 0.090 MiB 2 divtime = browser.find_by_css(var_y).first.value

514 99.910 MiB 0.000 MiB 2 divtime_2 = divtime.replace(' MYA', '')

515 99.910 MiB 0.133 MiB 6 missing = missing.append({'Taxa1' : taxon_a, 'Taxa2' :

taxon_b,

516 99.910 MiB 0.000 MiB 4 'Div.Time' : divtime_2}, ignore_index

= True)

517 99.910 MiB 0.000 MiB 2 time = tm.datetime.now()

518 99.910 MiB 0.000 MiB 2 print('{:<25}'.format(taxon_a), end="")

519 99.910 MiB 0.000 MiB 2 print('{:<25}'.format(taxon_b), end="")

520 99.910 MiB 0.000 MiB 2 print('{:<5}'.format(divtime_2), end="")

521 99.910 MiB 0.000 MiB 2 print(time)

522 99.910 MiB 0.000 MiB 1 output_name = input("File name (.csv) for missing values: ")

523 99.910 MiB 0.000 MiB 1 if output_name.endswith(".csv") is True:

524 99.910 MiB 0.000 MiB 1 pass

525 elif output_name.endswith('.csv') is False:

526 output_name = output_name + '.csv'

527 99.945 MiB 0.035 MiB 1 missing.to_csv(output_name, index=False)

528 else:

529 print("No missing values detected!")

530 100.078 MiB 0.000 MiB 2 elif choice == 'b':

531 99.945 MiB 0.000 MiB 1 infile_1 = input("File to check (.csv): ")

532 99.945 MiB 0.000 MiB 1 if infile_1.endswith('.csv') is True:

533 99.945 MiB 0.000 MiB 1 pass

534 elif infile_1.endswith('.csv') is False:

535 infile_1 = infile_1 + '.csv'

536 99.957 MiB 0.012 MiB 1 data = pd.read_csv(infile_1)

537 99.941 MiB -0.016 MiB 1 infile_2 = input("Missing value file (.csv): ")

538 99.941 MiB 0.000 MiB 1 if infile_2.endswith('.csv') is True:

539 99.941 MiB 0.000 MiB 1 pass

540 elif infile_2.endswith('.csv') is False:

541 infile_2 = infile_2 + '.csv'

542 99.953 MiB 0.012 MiB 1 missing = pd.read_csv(infile_2)

543 99.953 MiB 0.000 MiB 1 length = len(missing.index)

544 100.008 MiB 0.000 MiB 3 for i in range(0, length):

545 100.008 MiB 0.000 MiB 2 new_value = missing.iloc[i]['Div.Time']

546 100.008 MiB 0.055 MiB 2 data['Div.Time'].fillna(new_value,inplace=True, limit=1)

547 100.008 MiB 0.000 MiB 1 output_name = input("File name (.csv) for replaced values: ")

548 100.008 MiB 0.000 MiB 1 if output_name.endswith('.csv') is True:

549 100.008 MiB 0.000 MiB 1 pass

550 elif output_name.endswith('.csv') is False:

551 output_name = output_name + '.csv'

552 100.078 MiB 0.070 MiB 1 data.to_csv(output_name, index=False)

553 100.078 MiB 0.000 MiB 1 print('Done!')

554 100.078 MiB 0.000 MiB 1 print()

555 100.078 MiB 0.000 MiB 1 elif choice == 'c':

556 100.078 MiB 0.000 MiB 1 infile_1 = input("Input tree file in .nwk format: ")

557 100.078 MiB 0.000 MiB 1 tree = Phylo.read(infile_1, "newick")

558 100.082 MiB 0.004 MiB 1 Phylo.draw_ascii(tree)

559 elif choice == 'm':

560 main_loop()

561 99.297 MiB 396.570 MiB 4 @profile

562 def get_download_path():

563 """Returns the default downloads path for linux or windows"""

564 99.297 MiB 0.000 MiB 4 if os.name == 'nt':

565 99.297 MiB 0.000 MiB 4 sub_key = r'SOFTWARE\Microsoft\Windows\CurrentVersion\Explorer\Shell Folders'

566 99.297 MiB 0.000 MiB 4 downloads_guid = '{374DE290-123F-4565-9164-39C4925E467B}'

567 99.297 MiB 0.000 MiB 4 with winreg.OpenKey(winreg.HKEY_CURRENT_USER, sub_key) as key:

568 99.297 MiB 0.000 MiB 4 location = winreg.QueryValueEx(key, downloads_guid)[0]

569 99.297 MiB 0.000 MiB 4 return location

570 else:

571 return os.path.join(os.path.expanduser('~'), 'downloads')

572 97.535 MiB 97.535 MiB 1 @profile

573 def main_loop():

574 """The main loop of the script"""

575 while True:

576 99.684 MiB 295.953 MiB 3 choice = menu_choice()

577 99.684 MiB 0.000 MiB 3 if choice is None:

578 continue

579 99.684 MiB 0.000 MiB 3 elif choice == 'q':

580 99.684 MiB 0.000 MiB 1 print( "Exiting...")

581 99.684 MiB 0.000 MiB 1 break # jump out of while loop

582 98.680 MiB 0.000 MiB 2 elif choice == '*':

583 99.684 MiB 198.422 MiB 2 check_available()

584 elif choice == 'a':

585 div_times_sing()

586 elif choice == 'b':

587 div_times_batch()

588 elif choice == 'c':

589 evol_timeline()

590 elif choice == 'd':

591 time_tree()

592 elif choice == 'e':

593 citation()

594 elif choice == 'f':

595 validate()

596 else:

597 print("Invalid choice.")
