# Supplementary material for: PAReTT: A Python Package for the Automated Retrieval and Management of Divergence Time Data from the TimeTree Resource for Downstream Analyses
Source: J Mol Evol. 2023 Apr 20;91(4):502–13. doi: 10.1007/s00239-023-10106-3 (PMC10277261; doi:10.1007/s00239-023-10106-3)
Supplement: Supplementary file 2 — Supplementary file2 (DOCX 180 KB) [file 239_2023_10106_MOESM2_ESM.docx]

**SUPPLEMENTARY FIGURES**

**Sup. Figure 1:** Diagrammatic representation of the batch retrieval of divergence times using a list of species as input. The list is iterated through to compile the divergence times of every possible combination and output is given as a three-column vectorized matrix. The output can then be converted to a full matrix for statistical analyses of distance matrices. (Image created in BioRender.com)

**
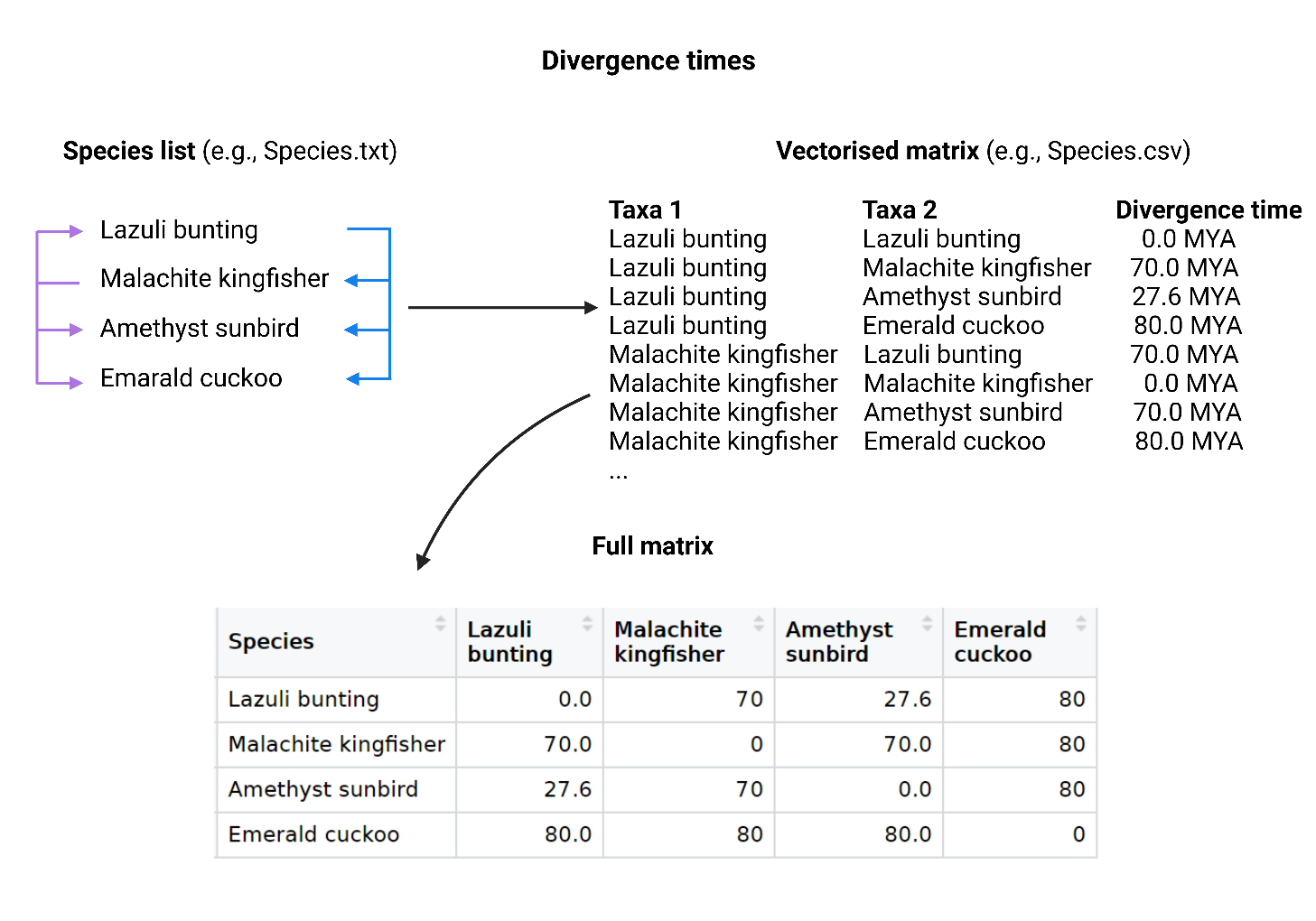
**
